# Clinicians’ Views on Using Artificial Intelligence in Healthcare: Opportunities, Challenges, and Beyond

**DOI:** 10.7759/cureus.45255

**Published:** 2023-09-14

**Authors:** Abdullah Alanazi

**Affiliations:** 1 Public Health and Health Informatics, King Saud Bin Abdulaziz University for Health Sciences, Riyadh, SAU; 2 Research, King Abdullah International Medical Research Center, Riyadh, SAU

**Keywords:** clinician-measured outcomes, technology, health data, electronic health records, artificial intelligence (ai)

## Abstract

Introduction: The healthcare industry has made significant progress in information technology, which has improved healthcare procedures and brought about advancements in clinical care services. This includes gathering crucial clinical data and implementing intelligent health information management. Artificial Intelligence (AI) has the potential to bolster further existing health information systems, notably electronic health records (EHRs). With AI, EHRs can offer more customized and adaptable roles for patients. This study aims to delve into the current and potential uses of AI and examine the obstacles that come with it.

Method: In this study, we employed a qualitative methodology and purposive sampling to select participants. We sought out clinicians who were eager to share their professional insights. Our research involved conducting three focus group interviews, each lasting an hour. The moderator began each session by introducing the study's goals and assuring participants of confidentiality to foster a collaborative environment. The facilitator asked open-ended questions about EHR, including its applications, challenges, and AI-assisted features.

Results: The research conducted by 26 participants has identified five crucial areas of using AI in healthcare delivery. These areas include predictive analysis, clinical decision support systems, data visualization, natural language processing (NLP), patient monitoring, mobile technology, and future and emerging trends. However, the hype surrounding AI and the fact that the technology is still in its early stages pose significant challenges. Technical limitations related to language processing and context-specific reasoning must be addressed. Furthermore, medico-legal challenges arise when AI supports or autonomously delivers healthcare services. Governments must develop strategies to ensure AI's responsible and transparent application in healthcare delivery.

Conclusion: AI technology has the potential to revolutionize healthcare through its integration with EHRs and other existing technologies. However, several challenges must be addressed before this potential can be fully realized. The development and testing of complex EHR systems that utilize AI must be approached with care to ensure their accuracy and trustworthiness in decision-making about patient treatment. Additionally, there is a need to navigate medico-legal obligations and ensure that benefits are equitably distributed.

## Introduction

The healthcare sector has significantly transformed toward enabling digitally driven solutions to aid the clinical and administrative decision-making process. The journey began with digital records, moved on to electronic medical records, and now we have intelligent electronic health record (EHR) systems [1]. These EHR systems are increasingly being interfused with advanced technologies, such as the Internet of Things (IoT), machine learning, blockchain, and artificial intelligence (AI), to optimize healthcare operations [2]. AI encompasses a range of technologies that empower machines to mimic human cognitive abilities [3]. AI technologies have been devised to analyze diverse health data types, including patient, behavioral, environmental, clinical, and pharmaceutical data. In addition, AI technologies have found extensive use in analyzing clinical data, such as medical images, EHRs, and physiological signals [4].

EHRs constitute comprehensive, constantly updated patient health records synchronized across multiple departments, providing an up-to-date compilation of complete patient records. EHRs represent a real-time, cumulative dataset of all patient medical records, including various providers, test labs, imaging labs, and pharmacies. The evolution of EHRs is a subject of immense interest within the healthcare industry. As we look toward 2030 and beyond, EHR systems are anticipated to transition from static records to dynamic, interactive engagement systems [5]. EHRs currently exhibit a highly structured format, but future iterations are expected to adopt a more flexible, responsive, and adaptive hierarchical model. Furthermore, the transactional nature of EHR will evolve into a more conversational approach, fostering more significant interactions between healthcare providers and patients [6]. This transformation would render EHR systems more accessible and user-friendly, enhancing patient experiences [7].

Integrating AI with EHR systems holds the potential to revolutionize the healthcare sector. By harnessing AI, EHR systems could significantly reduce error probabilities and augment the quality of patient care. Several AI features, such as natural language processing (NLP), predictive analytics, and machine learning, can be incorporated into EHR systems [8]. Each feature can enhance the efficacy and efficiency of EHR systems, thereby contributing to improved patient outcomes. Despite the significant strides made in healthcare due to the integration of AI, it is vital to recognize that AI cannot wholly supplant human clinicians due to its inherent limitations [9]. In addition to that, physicians needed to gain more familiarity with AI in general and particularly the applications of AI in medicine [10,11]. One study revealed that physicians have heard about AI very few times, and only 3% have attended courses in AI [10]. Although physicians are optimistic about the future of AI, only 6% are familiar with AI [11]. Hence, the reliability and validity of AI to improve healthcare is still doubtful [12].

Consequently, it is crucial to identify the areas of healthcare delivery that AI will influence from care providers’ perspectives. Regrettably, current scientific literature and public policy discussions need to address the integration of AI into healthcare delivery. This article aims to provide a comprehensive overview of this critical matter. It discusses how AI has been and will continue to act as a transformative force in enhancing healthcare delivery.

## Materials and methods

This a qualitative study in which focus group meetings were conducted to solicit data to address the purposes of the study. The study employed a purposive sampling strategy to collect data on attitudes toward AI-enabled healthcare delivery, with clinicians as participants. It is recommended to have a sample size of 20-30 individuals for a qualitative study, provided that population integrity is maintained during recruitment. All participants were selected based on their willingness to share their perspectives and signed a consent form. Communication was conducted exclusively in English. No compensation was offered for their time. The moderator initiated each focus group meeting to foster participation by explaining the study's objectives and assuring confidentiality. The investigator has led a balanced and inclusive discussion. This is accomplished by formulating and expressing ideas, presenting evidence, and receiving feedback. Our qualitative study involved interviewing a diverse range of clinicians to obtain a breadth of opinions, also called "triangulation of subjects." This approach would help us avoid biased responses by diversifying respondents. The semi-structured rounds cover a set of questions while allowing flexibility. Participants were encouraged to elaborate freely, combining contributions from all and assuring a planned framework for each session. The investigator used open-ended questions and prompts to explore various AI-related topics. The participants were instructed to introduce themselves, providing their gender, age, background, experience, and comfort level with information technology. Participants were asked about their familiarity with AI and EHR technologies and their opinions on their potential applications and short- and long-term impacts. They were also asked about potential barriers and threats to the broader adoption of AI technology in healthcare and the missed opportunities if it is not implemented.

We conducted a collaborative study with ethical considerations, obtaining approval from the Institutional Review Board of King Abdullah International Medical Research Center (approval no. IRB/1740/23) beforehand. The participants were assured of confidentiality, voluntary participation, and the ability to withdraw from the study at any time.

## Results

The data provided a thorough overview of the interview participants, representing various health specialties and experience levels. Notably, 54% of the participants were female. Most participants indicated they had experience with extracurricular EHR use in inpatient and ambulatory clinic settings. Additionally, most participants expressed moderate to high comfort with EHR usage. For further details on the socioeconomic characteristics of the participants, please refer to Table 1.

**Table 1:** Socioeconomic characteristics of the participants

Measure	Item	Frequency	Percentage %
Gender	Male	12	46.2
	Female	14	53.8
Clinical Experience	< 5	6	23.1
	5-9	13	50
	10-14	7	26.9
Specialty	Physicians	3	11.5
	Dentists	5	19.2
	Nurses	4	15.4
	Pharmacists	3	11.5
	Laboratory specialists	3	11.5
	Radiology specialists	3	11.5
	Other	5	14.4
Extracurricular EHR experience	Extracurricular training	4	15.4
	Inpatient setting	8	30.8
	Ambulatory Clinic	14	53.8
Comfort levels with EHR	Low	3	11.5
	Moderate	10	38.5
	High	13	50

The participants highlighted various advantages of integrating AI into EHR. For instance, one physician mentioned, "The integration of AI into EHRs has streamlined the extraction and analysis of detailed data, thereby facilitating the development of personalized treatment recommendations and ultimately leading to improved patient outcomes."

Another participant claimed, "The healthcare organization has been transformed by incorporating AI technologies into their process, clinically and administratively. AI technology has enhanced the efficiency of patient data extraction, analysis, and treatment recommendations by aiding decision-making processes." Another participant noted, "With emerging applications of AI in medical imaging technology and diagnostic screenings, there has been an unprecedented enhancement in patient care." Furthermore, a participant mentioned, "The integration of AI technology has significantly improved healthcare. It has made patient record management more efficient, boosted diagnostic accuracy, and allowed physicians to devote more time to patient care."

Our research identified five prominent areas of AI applications in EHR in the short term. These areas comprise clinical decision support systems, predictive analysis, data visualization, NLP, patient monitoring, mobile technology, and telemedicine encountering future trends. For the long term, the participants identified the following applications: precision medicine, robotic surgeries, drug discovery and development, population health management, and the introduction of artificial organs. Table 2 demonstrates the applications of AI-enabled EHR from short- and long-term perspectives.

**Table 2:** Short- and long-term applications of AI-enabled healthcare delivery AI: artificial intelligence, NLP: natural language processing

Short-term and long-term AI-enabled healthcare delivery		No of participants (%)
Short term	Decision support systems	25 (96%)
	Predictive analysis	23 (88.5%)
	Data visualization	22 (84.6%)
	NLP	16 (61.5%)
	Patient monitoring	16 (61.5%)
Long term	Precision medicine	22 (84.6%)
	Mobile technology and telemedicine	17 (65.4%)
	Robotic surgeries	14 (53.8%)
	Drug discovery and development	12 (46%)
	Population health management	11 (42.3%)

We have identified several sub-themes within each core theme and provided illustrative quotes for clarity. We delved into each area to comprehensively understand the topic. Further, the challenges of the intelligent EHR are demonstrated in Table 3. One participant stated "The integration of AI technology into the healthcare industry has the potential to bring numerous advantages. However, it is important to acknowledge that this shift also comes with challenges that must be carefully considered and addressed." Another participant reveals "The use of AI in healthcare has the potential to bring about significant changes. However, many are worried about safety and ethical issues. There is a risk that AI could further perpetuate biases and inequalities in the healthcare industry. Healthcare professionals, AI experts, and policymakers must work together closely." A third participant expressed her concern as the fellows: "The field of AI faces challenges such as biased data, lack of transparency, and governance. Biased data can lead to unfair outcomes, while a lack of transparency and regulation can result in AI misuse. Proper governance is crucial for ethical and responsible AI use that does not harm society." The challenges are grouped and presented in Table 3 for AI-enabled healthcare delivery short- and long-term challenges.

**Table 3:** Short- and long-term challenges of AI-enabled healthcare delivery AI: artificial intelligence

Short-term and long-term challenges of AI		No of participants (%)
Short term	Data quality concerns	25 (96%)
	Ethical and privacy concerns	24 (92.3%)
	Lack of trust and transparency bias	19 (73.1%)
	Technical limitations	18 (69.2%)
	Workforce displacement	14 (53.4%)
Long term	Ethical and philosophical concerns	23 (88.5%)
	Dependence on AI	19 (73.1%)
	Cybersecurity risks	18 (69.2%)
	Cost and resource allocation	16 (61.5%)
	Unintended consequences of AI	13 (50%)

Table 4 illustrates the threats of not implementing AI in healthcare from short- and long-term perspectives.

**Table 4:** Short- and long-term threats of implementing AI AI: artificial intelligence

Threats of not implementing AI-enabled healthcare delivery		No of participants (%)
Short term	Lack of efficiency and productivity	20 (76.9%)
	Missed diagnoses and treatment opportunities	12 (46.1%)
	Inability to provide personalized treatments	10 (38.5%)
	Increases in healthcare costs	7 (26.9%)
	Limitations of medical research	5 (19.2%)
Long term	Increased healthcare costs	14 (53.8%)
	Delayed access to cutting-edge technology	9 (34.6%)
	Missed precision medicine opportunities	7 (26.9%)
	Loss of research capabilities	7 (29.6%)
	Limited healthcare access and outcomes	4 (15.4%)

Interestingly, the participants revealed no differences in perspective, irrespective of their specialization, years of experience, or comfort level with EHR.

## Discussion

The healthcare industry faces several hurdles, including escalating healthcare costs and a shortage of healthcare professionals [13]. To address these issues, the industry is exploring modern solutions to enhance healthcare services while curbing expenditure. One promising solution involves employing advanced information technology, specifically AI, which has made significant strides in simulating human cognitive functions and processing massive data volumes [14]. However, users of AI, clinicians in this context, are not fully aware or familiar with the applications of AI in medicine. Two studies showed that only 23% and 27% of doctors are aware of using AI in medicine, indicating a low level of knowledge. However, doctors in both studies positively perceived AI in medicine [15,16]. Similarly, around 80% of doctors disclosed a lack of knowledge about AI applications, and 83% stated no knowledge about the limitations of AI in medicine; nonetheless, the majority of them, 65%, believed that AI would improve their careers [17].

Although some studies revealed that doctors have positive attitudes toward AI in principle, it showed that doctors were reluctant to use AI in their clinics and were cautious when they were asked about the impact of AI on their patients [18,19].

Our study explored clinicians' views on the potential and obstacles of the next-generation EHR with the expansion of using AI. It is assumed that the roles of physicians would be transformed and consequently impact the physician-patient relationship [20]. Therefore, it is time to prepare physicians for the next move, as their involvement and contribution are required. A sole machine-patient relationship is impossible, as patients always need human physicians [21,22].

Our research has shown that AI-enabled healthcare delivery in the future should incorporate several critical applications such as decision support systems, predictive analysis, data visualization, NLP, patient monitoring, mobile technology, and other applications. However, these intelligent systems also pose certain challenges, such as the hype surrounding AI and its future applications, the quality of clinical data, unclear decision-making processes, user resistance, technical limitations, and medico-legal issues. These findings can help us comprehend the potential and limitations of AI-powered healthcare systems and pave the way for further research in this field. Similar research conducted by Sunarti et al. found that AI holds tremendous promise for enhancing patient diagnostics, prevention, and treatment. Additionally, AI can improve cost efficiency and promote equality in healthcare services [23], in addition to its impact on drug development, genomics, and public health management [24].

Despite these benefits, the public sector has yet to adopt AI technology widely. Some concerns around patient privacy and autonomy have emerged as potential challenges when implementing AI applications [23]. Further, Mahajan et al. pointed out the potential of AI to assist in early detection and diagnosis, which is a significant benefit to society. To ensure a positive impact, it is recommended that we refer to it as "augmented Intelligence." However, addressing ethical concerns and prioritizing collaboration is crucial to ensure proper implementation. Mahajan et al. also acknowledged that AI could diagnose certain medical conditions [25]. However, it is essential to consider potential risks such as automation bias, over-reliance, data privacy concerns, algorithmic biases, and corrigibility issues [26].

Below, we explored various themes about AI opportunities and challenges during the upcoming discussion as per the responses of the study's participants. Additionally, we provided further explanations of the related studies that exist.

Opportunities for AI

According to the participants, AI holds the potential to impact healthcare delivery in various aspects. However, the scope of applications of AI is limited to cases that are not complex, fit the predictive model, and do not require emotional or human aspects, like end-of-life and breaking bad news [27].

The healthcare sector continuously seeks new techniques and equipment to improve clinical procedures through clinical decision support systems (CDSS), as revealed by 96% of the participants. Reminders, suggestions, messaging platforms, decision support tools, treatment planning, and clinical workflow services are just a few of the many tactics used in these strategies [28]. For more than 50 years, CDSS have been proposed to help healthcare professionals make educated decisions and advance patient care for diagnosis and treatment [29]. In a recent article, the use of fuzzy decision support systems for diagnosing diabetes is explored. Due to their accuracy, dynamic nature, semantic intelligence, and interpretability, these systems provide distinct advantages over conventional CDSS implementation [30]. Researchers created a novel technique to identify inflammatory syndrome in individuals with comorbid illnesses and viral infections in a different study. They researched to ascertain the clinical metric performance of a cloud-based computerized sepsis CDSS in addition to developing and implementing a computerized early warning tool for sepsis [31]. The study's objectives were to comprehend the epidemiology of sepsis and find areas for quality enhancement. A Babylon AI triage and diagnostics system was tested in another study, providing more accuracy and safety than human doctors when providing triaging advice [12].

Predictive analytics in healthcare can significantly influence life-saving efforts [5]. This is mentioned by 88.5% of the participants. Implementing AI in healthcare allows for intricate pattern detection and relationship identification within the data, thereby reducing the need for additional healthcare professionals to analyze data, especially in the era of big data. Integrating a robust AI system with a reliable EHR system allows potential health risks to be identified, health conditions evaluated, and appointments automatically scheduled [12].

Emergency services can be alerted promptly to facilitate life-saving interventions [32]. The application of AI with EHR data has demonstrated success in various medical fields. In cardiology, AI techniques have been used to detect heart failure early, predict the onset of congestive heart failure, and enhance risk assessment for patients with suspected coronary artery disease [33]. Similarly, in ophthalmology, AI has been used with EHR data to predict risks associated with cataract surgery, improve glaucoma and age-related macular degeneration diagnoses, and assess diabetic retinopathy risk [34]. However, the reuse of EHR data presents challenges due to its complexity and heterogeneity. Interpreting this data necessitates strategies such as information extraction, dimension reduction, and predictive modeling, which are typical of machine learning and AI techniques [35]. Challenges also exist in AI's application for medical images and biosignal analysis. Medical image analysis heavily depends on deep learning architectures, which require extensive data for training [36]. This can be a challenge in rare diseases where little data is available for training [37]. Additionally, physiological signal analysis is computationally expensive and can be influenced by noise and measurement errors [38]. Developing AI approaches that are resistant to noise is essential for analyzing physiological data acquired by consumer-grade devices of lower quality [39].

Several studies suggested that how medical information is presented (data visualization) can significantly impact decision-making, and this was revealed by 85% of the participants. A study conducted before the widespread implementation of EHRs demonstrated that data display significantly influences physician decision-making regarding clinical trials [40]. On the other hand, another study found that presenting information in charts rather than tables improved physicians' ability to identify outliers [41]. Recent research showed that healthcare providers can more efficiently identify trends and make decisions when laboratory data are presented in graph form [42]. The advent of interactive data visualization has enabled improved data insight through visualizations and interaction. Over the past few years, several EHR-based interactive data visualizations have been developed to aid healthcare providers in performing data-driven tasks and activities. Lesselroth et al. surveyed various visualization techniques for EHRs [43].

Integrating NLP with medical expert systems and chatbots can enhance healthcare services, and this role was demonstrated by 61.5% of the participants. NLP can extract symptoms, diagnoses, and treatments from medical records through practical symptom analysis [44]. This information can then be used to train medical expert systems, aiding doctors in diagnosing diseases more accurately. Using NLP, chatbots that educate and help patients can also be created. The following are some advantages of combining NLP with chatbots and medical expert systems: better diagnosis by extracting data from medical records, NLP can help clinicians make more accurate diagnoses, resulting in early detection and treatment, which improves patient outcomes [45].

Additionally, NLP can create chatbots that give patients individualized information and support, assisting them in managing their care and understanding their condition [46]. NLP can also assist in lowering healthcare expenses by automating operations like information extraction from medical records, patient support, and information delivery [44]. Some issues must be resolved before NLP's full promise in healthcare can be realized. One difficulty is requiring substantial amounts of high-quality data for accurate NLP model training. The gathering and compilation of medical data are complicated because it is frequently complex and unstructured. The requirement for a careful assessment of NLP models presents another difficulty. NLP models are susceptible to bias, which might result in incorrect diagnoses or suggestions. A thorough evaluation is essential to guarantee the fairness and correctness of NLP models [47]. Combining NLP with chatbots and medical expert systems can improve healthcare despite these obstacles.

The advancement of AI technology has dramatically impacted the healthcare industry. It has improved patient engagement and ensured continuity of care. Patient monitoring has undergone substantial progress. Therefore, 61.5% of the participants mentioned the role of AI applications. With AI-powered technologies, medical personnel may gather extensive patient vital sign data, including sleep patterns, heart rate, blood pressure, and other vital metrics [48]. This technology has shown great use in hospitals, where it monitors patients' cardiovascular and respiratory data, analyzes data, and provides individualized health and pharmaceutical advice. Setting up follow-up visits and ensuring patients receive care immediately help improve drug adherence. In order to monitor patients' health and make individualized dietary and medication recommendations, wearable sensors are also used. These recommendations are based on the information gathered by the sensors, allowing doctors to provide individualized medical advice. Despite the advantages of AI in this regard, addressing security and privacy issues raised by this technology is critical [49]. These systems must safeguard the personal data they acquire and keep it private from unauthorized users. Medical professionals must fully utilize the technology while minimizing associated hazards as they continue investigating AI's possibilities in healthcare.

Additionally, there are other areas of application and future trends. AI can trigger, notify, and recommend patient-specific care in addition to the uses mentioned above, which were mentioned by 85% of the participants. For instance, AI can offer recommendations for medicines, a particular course of treatment or approach, and suggestions for public health precautions and preventative measures [50]. AI can improve the effectiveness of the healthcare system in addition to patient monitoring and individualized care. AI can help to guide surgical robots, helping to improve precision and accuracy during surgeries; this was disclosed by 54% of the participants. This can result in fewer complications and faster recovery times for patients. One study found that smart tissue autonomous robots outperformed surgeons in controlled trial experiments; at least, they can be used to augment surgeons' capabilities [51]. Forty-six percent of the participants envisioned the role of AI in drug discovery and development. AI can be used to identify potential drug targets and perform more efficient drug screening, leading to the development of new treatments and therapies. One study anticipates that AI can revolutionize the pharmaceutical industry, as it can improve the life cycle of a drug from discovery until development across the clinical trial phases, including post-marketing surveillance [52].

The final theme of AI applications was the role of AI in population health management, which was disclosed by 42% of the participants. AI can identify trends and patterns in the health of entire populations, allowing healthcare providers to develop proactive interventions and preventive measures. AI-enabled EHR can detect cases with particular importance for public health from a text in the patient chart of international classification of disease codes through utilizing NLP. Furthermore, AI can help establish and optimize disease surveillance systems [53]. Other potential uses of AI are found in the literature. AI-powered systems can automate scheduling appointments, processing insurance claims, and managing patient records, freeing time for healthcare providers to focus on patient care. Overall, AI holds the potential to significantly impact healthcare, improving patient care, reducing costs, and enhancing the efficiency of the healthcare system.

Challenges of AI

As the healthcare industry moves toward integrating AI technology, it is essential to acknowledge that this shift comes with challenges. Many participants expressed concerns regarding the nature of the healthcare process and decision-making. In addition to the generic challenges facing AI in all industries, healthcare has its distinct nature, the operationalization of the concept of health and sickness, the liability that is usually regulated by medicine and civil law, the complexity of environment and practice, confidentiality and data privacy, and the fear of a technological singularity, the moment that AI surpassed human intelligence [10,24,53].

One of the significant challenges stated by 96% of the participants that must be addressed is the quality of today's healthcare data. As many healthcare providers know, this data can be challenging to come by due to its sourcing from various platforms. These platforms may include insurance databases, pathology systems, EHRs, and medical imaging archives, all of which may have different systems and structures. As we continue to explore the potential of AI in the healthcare industry, it is imperative that we efficiently collect and process data with optimal quality to unlock its benefits fully. The accuracy of AI systems is contingent upon the data quality used for their training. Any inaccuracies or biases present within the data can result in significant disparities in system accuracy, mainly when applied to populations that differ from the original training data. For instance, an AI system trained on data from a predominantly White population may be less effective when utilized on individuals of different ethnicities [54].

Most participants (92%) showed concerns regarding patient privacy and data confidentiality when using AI medical and legal contexts. Integrating AI systems in healthcare has raised concerns regarding privacy, equity, security, and benefits. These systems are designed to collect and analyze personal information, including sensitive medical, genetic, and lifestyle data. The public is concerned about how this information will be utilized and safeguarded. Should an AI system make predictions about a patient's health, it could lead to discrimination or denial of access to insurance or employment opportunities. Furthermore, with AI involvement in healthcare services, regulatory and legal authorities must collaborate with healthcare providers and software developers to establish clear lines of responsibility and address questions about accountability for errors. This clarification is necessary to ensure accountability for system use and users regarding medico-legal obligations in decision-making [55].

Although AI can transform healthcare, it is crucial to exercise caution, given the exaggerated claims surrounding AI and the fact that the technology is still in its early stages [56]. Technical limitations related to human vision, language processing, and context-specific reasoning must be addressed, as this was mentioned by 69% of the participants. One of the main challenges is the need for high-quality engineering practices and evidence standards. The primary hurdle revolves around integrating AI into existing healthcare systems, which is further complicated by vendors who offer stand-alone solutions or limit their focus to specific areas of care [57]. EHR systems that use AI are intricate and need careful development and testing. Some EHR companies are early in incorporating AI functionalities into their products beyond just rule-based clinical decision support [58]. In order to ensure that healthcare providers are equipped with the best tools, we must prioritize accuracy and reliability when it comes to AI systems. These systems must be designed with a focus on serving the needs of humans, and they must be held to high standards of quality and performance. This concern was raised by 73% of the participants. Schwalbe et al. thoroughly analyzed the current state of research in the field. Their work identified eight key concerns that must be addressed to assess the trustworthiness of AI systems in healthcare. They also examined the various standards to ensure AI policies' legitimacy, morality, and robustness [59].

Any inaccuracies or biases present within the data can result in significant disparities in system accuracy, mainly when applied to populations that differ from the original training data. For instance, an AI system trained on data from a predominantly White population may be less effective when utilized on individuals of different ethnicities. By taking these steps, we can ensure that AI is utilized safely and effectively to improve healthcare outcomes for all. To achieve this objective, it is necessary to create ethical guidelines for AI use in healthcare, train AI systems on data representative of the population, and develop and enforce strong privacy and security regulations for AI systems in healthcare [60].

Additionally, it is essential to ensure that AI-powered medical devices are affordable for everyone to prevent a two-tiered healthcare system. The concern about the cost and the needed resources is mentioned by 61% of the participants. Additionally, AI systems are susceptible to cyber-attacks and data breaches that can compromise sensitive patient information. This serious concern was raised by 69% of the participants as a long-term challenge to incorporate AI in healthcare. Such occurrences can have severe consequences for patients, including financial loss, identity theft, and discrimination. As such, healthcare professionals and stakeholders must consider these factors when implementing AI technologies in healthcare settings. By discerning potential biases and vulnerabilities within AI systems, we can optimize their benefits while minimizing risks. To address these concerns, it is critical to establish robust privacy and security regulations for AI systems in healthcare.

Next, the participants anticipated how not implementing AI would impact their present and future practices. Most participants disclosed concerns about losing the opportunity to enhance efficiency and productivity, as the cost of services is expected to escalate soon. Not adopting AI may delay significant advancements in precision medicine, leading to missed opportunities to improve health outcomes for individuals. Overall, the lack of adoption of AI can lead to insufficient data to develop healthcare innovations like telemedicine, remote monitoring, and IoT diagnostic tech, which can limit access to healthcare and reduce overall health outcomes.

Recommendations

Healthcare professionals play an active role in their integration process. Their involvement will provide necessary oversight and validation, guaranteeing that the technology is clinically plausible but also transparent and valid. Adopting AI must be carefully managed and monitored to empower all stakeholders to participate. Only then can we ensure that AI technology is utilized to its fullest potential to improve patient care [56]. AI technology and cognitive ergonomics can simplify the use of medical tools for practitioners. However, there needs to be more guidelines for designing graphical user interfaces for advanced AI systems. This is concerning since doctors' heavy workloads can impact patient safety and well-being. Unfortunately, the healthcare industry has yet to evaluate AI technology from a cognitive ergonomics perspective [61]. In order to guarantee the dependability and efficiency of AI systems within the healthcare industry, it is crucial to educate clinicians about the potential and limitations of AI [19]. Based on our findings, the fourth recommendation is to set a governing body to regulate and oversee the data life cycle in the hospital, including ethical guidelines, policies to ensure preserving patient privacy, and proper strategies to ensure the safety of data and the management systems.

At the end of our study, this is a qualitative study, and the purpose is to explore the clinicians' perspectives. Further studies should target approaches for more confirmatory results by assessing the impact of AI interventions on physicians' practice and patient outcomes.

## Conclusions

The healthcare industry is evolving, and AI technology integration in healthcare delivery is diverse and vast, ranging from advanced decision support systems, predictive analysis, and data visualization to natural language processing and mobile technology. Other uses of AI in healthcare include patient monitoring, precision medicine, robotic surgeries, drug discovery and development, and population health management. However, to achieve this, several challenges need to be addressed. These include the assurance of data quality, the mitigation of ethical and privacy concerns, the establishment of trust and transparency, the overcoming of technical limitations, the mitigation of bias, the management of workforce displacement, the handling of cybersecurity risks, and the optimization of cost and resource allocation. Consequently, incorporating technology into the healthcare industry is a collaborative effort that necessitates the participation of healthcare professionals. Their input is vital to guarantee that the technology is clinically viable and transparent. Our findings indicate that establishing a regulatory body to oversee data life cycles is advisable to achieve this goal. This governing entity should create ethical protocols and policies to safeguard patient privacy and implement effective strategies to maintain the security of data and management systems.
